# Authors' reply

**Published:** 2010

**Authors:** Ivana L Romero, João B N S Malta, Cely B Silva, Lycia M J Mimica, Kaz H Soong, Richard Y Hida

**Affiliations:** 1 Department of Ophthalmology, Santa Casa de São Paulo, São Paulo, Brazil; 2 Department of Microbiology, Santa Casa de São Paulo, São Paulo, Brazil; 3 W.K. Kellogg Eye Center, University of Michigan, Ann Arbor, MI, U.S.A; 4 Department of Ophthalmology, Keio University – School Medicine, Tokyo, Japan

Dear Editor,

We appreciate the opinions about our article[1] by Wiwanitkit.[2] The adhesives, mainly ethyl-cyanoacrylate, have been studied by our group in different features.[3,4] Previously, we mentioned that “high temperatures related to exothermic reaction of ethyl-cyanoacrylate polymerization may possibly induce a temporary bactericidal effect”[3] like Dr Viroj Wiwanitkit cited very well in his letter to the editor. Other studies, however, suggest that the antimicrobial activity could be related to the degradation products.[5,6] Formaldehyde, one of degradation products of cyanoacrylate, for example, destroys cell membranes by denaturing the membrane proteins.[7] Toxic degradation products of cyanoacrylate may further slow cell growth, damage its physiological needs, and change its cellular membrane structure by inhibiting a specific receptor.[8]
